# Altered Nitric Oxide Bioavailability Contributes to Diesel Exhaust Inhalation‐Induced Cardiovascular Dysfunction in Man

**DOI:** 10.1161/JAHA.112.004309

**Published:** 2013-02-22

**Authors:** Jeremy P. Langrish, Jon Unosson, Jenny Bosson, Stefan Barath, Ala Muala, Scott Blackwell, Stefan Söderberg, Jamshid Pourazar, Ian L. Megson, Andrew Treweeke, Thomas Sandström, David E. Newby, Anders Blomberg, Nicholas L. Mills

**Affiliations:** 1 University/British Heart Foundation Centre for Cardiovascular Science, University of Edinburgh, Edinburgh, UK (J.P.L., D.E.N., N.L.M.); 2 Department of Public Health and Clinical Medicine, Division of Medicine/Respiratory Medicine, Umeå University, Umeå, Sweden (J.U., J.B., S.B., A.M., J.P., T.S., A.B.); 3 Department of Clinical Biochemistry, Royal Infirmary, Glasgow, UK (S.B.); 4 Department of Public Health and Clinical Medicine, Division of Medicine/Cardiology, Umeå University, Umeå, Sweden (S.); 5 Free Radical Research Facility, UHI Department of Diabetes and Cardiovascular Science, Inverness, UK (I.L.M., A.T.)

**Keywords:** air pollution, endothelial function, nitric oxide, nitric oxide synthase, vascular biology

## Abstract

**Background:**

Diesel exhaust inhalation causes cardiovascular dysfunction including impaired vascular reactivity, increased blood pressure, and arterial stiffness. We investigated the role of nitric oxide (NO) bioavailability in mediating these effects.

**Methods and Results:**

In 2 randomized double‐blind crossover studies, healthy nonsmokers were exposed to diesel exhaust or filtered air. *Study 1*: Bilateral forearm blood flow was measured during intrabrachial infusions of acetylcholine (ACh; 5 to 20 μg/min) and sodium nitroprusside (SNP; 2 to 8 μg/min) in the presence of the NO clamp (NO synthase inhibitor N^G^‐monomethyl‐l‐arginine (l‐NMMA) 8 μg/min coinfused with the NO donor SNP at 90 to 540 ng/min to restore basal blood flow). *Study 2*: Blood pressure, arterial stiffness, and cardiac output were measured during systemic NO synthase inhibition with intravenous l‐NMMA (3 mg/kg). Following diesel exhaust inhalation, plasma nitrite concentrations were increased (68±48 versus 41±32 nmol/L; *P*=0.006) despite similar l‐NMMA–induced reductions in basal blood flow (−20.6±14.7% versus −21.1±14.6%; *P*=0.559) compared to air. In the presence of the NO clamp, ACh and SNP caused dose‐dependent vasodilatation that was not affected by diesel exhaust inhalation (*P*>0.05 for both). Following exposure to diesel exhaust, l‐NMMA caused a greater increase in blood pressure (*P*=0.048) and central arterial stiffness (*P*=0.007), but reductions in cardiac output and increases in systemic vascular resistance (*P*>0.05 for both) were similar to those seen with filtered air.

**Conclusions:**

Diesel exhaust inhalation disturbs normal vascular homeostasis with enhanced NO generation unable to compensate for excess consumption. We suggest the adverse cardiovascular effects of air pollution are, in part, mediated through reduced NO bioavailability.

**Clinical Trial Registration:**

URL: http://www.ClinicalTrials.gov. Unique identifiers: NCT00845767 and NCT01060930.

## Introduction

Exposure to air pollution increases cardiovascular morbidity and mortality,^1–4^ and is associated with the triggering of acute myocardial infarction.^5^ Recent controlled exposure studies in man have shown that inhalation of dilute diesel exhaust, at environmentally relevant concentrations, leads to an impairment of both vascular vasomotor and fibrinolytic function.^6^ Similarly, exposure to concentrated ambient particles causes acute arterial vasoconstriction^7^ and an increase in diastolic blood pressure.^8^ Although providing plausible explanations for the increase in cardiovascular events,^9^ the mechanisms of how air pollution induces these cardiovascular abnormalities remain to be established.

Inhalation of fine particulate matter causes inflammation within the lungs,^10–11^ in part, through local oxidative stress.^12–13^ Vascular oxidative stress may subsequently develop as a consequence of the systemic response to pulmonary inflammation and oxidative stress, or through reactive oxygen species generation within the vascular wall following translocation of particles into the circulation.^14–15^ Irrespective of the site of generation, it has been proposed that this oxidative stress has a direct influence on the function of the vascular endothelium^16^ as well as reducing the vascular bioavailability of nitric oxide (NO).^6,17^

NO is a powerful endogenous vasodilator and plays a crucial role in the maintenance of basal vascular tone.^18^ In patients with pre‐existing vascular endothelial dysfunction, such as those with hypercholesterolemia, stimulated endothelium‐dependent NO‐mediated vasodilatation is impaired, and this is thought to be mediated through oxidative stress‐mediated depression of NO bioavailability.^19^ In rats exposed to inhaled particles, the generation of oxygen‐centered free radicals leads to the scavenging of NO, thereby reducing its bioavailability.^20^

In this study, we investigated the hypothesis that changes in NO bioavailability contribute to the cardiovascular dysfunction induced by diesel exhaust inhalation.

## Methods

### Subjects

Thirty‐three healthy nonsmoking volunteers were recruited from the Department of Medicine, Division of Respiratory Medicine and Allergy, University Hospital, Umeå, Sweden. Subjects taking regular medication (excluding the oral contraceptive pill), with a significant occupational exposure to air pollution or those with intercurrent illness were excluded. All subjects were free of symptoms of respiratory tract infection for at least 6 weeks leading up to the study. The trial was performed with the approval of the local research ethics committee, in accordance with the Declaration of Helsinki, and with the written informed consent of each participant.

### Diesel Exhaust Exposure

Diesel exhaust was generated using an idling Volvo diesel engine (Volvo TD40 GJE, 4.0 L, 4 cylinders, 680 rpm). Over 90% of the exhaust was shunted away, the remaining part diluted with high‐efficiency particulate air (HEPA) filtered air and fed into the exposure chamber at steady state concentration. During the exposure, air was sampled in the breathing zone of the subjects for oxides of nitrogen, particle mass concentration and total hydrocarbons (measured as propane). Filter samples were collected and analyzed gravimetrically to determine mass concentration. The exposures were standardized based on particulate matter (particles with a mean aerodynamic diameter ≤10 μm [PM_10_]) concentrations and a mean airborne particle concentration of 313±11 μg/m^3^ was generated. The particle exposure was associated with concentrations of nitrogen dioxide (NO_2_) of 0.9±0.1 ppm, NO of 3.1±0.2 ppm and total hydrocarbons of 2.5±0.2 ppm. Temperature and humidity in the chamber were controlled at 22°C and 50%, respectively.

### Study Design

Subjects attended on 2 occasions at least 1 week apart. In 2 randomized double‐blind crossover studies, subjects were exposed to either filtered air or dilute diesel exhaust at ≈300 μg/m^3^ in a specially built diesel exposure chamber as described previously.^21^ During the exposures, subjects performed 15‐minute periods of exercise on a bicycle ergometer, to achieve a mean minute ventilation of 20 L/min per m^2^ as determined by a prestudy exercise test, alternating with 15 minutes of rest.

Based on previous studies, vascular assessments were performed 2 hours after the exposure.^6,22^ All subjects abstained from alcohol for 24 hours, caffeinated drinks for 8 hours and food for 4 hours prior to the cardiovascular assessments, and remained indoors between the exposure and assessments to minimize additional exposure to air pollution. Venous blood samples were collected for measurement of plasma nitrite concentration, endogenous NOS inhibitors, and total and differential cell counts. Lung function was determined by spirometry at baseline and 2 hours following the exposure (Jaeger Masterlab, Jaeger AG) recording forced vital capacity (FVC), forced expiratory volume in 1 second (FEV_1_), and slow vital capacity (VC).

### Study 1: Local NO Synthase Inhibition

Eighteen subjects were recruited into this study. Forearm blood flow was determined using forearm venous occlusion plethysmography and detected using mercury‐in‐silastic strain gauges placed around each forearm as described previously.^23^ All subjects underwent unilateral brachial artery cannulation using a 27‐gauge steel needle (Coopers Needle Works Ltd) under controlled conditions. After a 30‐min baseline infusion of 0.9% saline, subjects received an infusion of the NO synthase (NOS) inhibitor N^G^‐monomethyl‐l‐arginine (l‐NMMA; Clinalfa^®^ basic, Bachem) at incremental doses of 2, 4, and 8 μmol/min. The infusion of l‐NMMA was continued at 8 μmol/min for the remainder of the study to fully inhibit all basal NOS activity.^24–25^ Sodium nitroprusside (SNP), an NO donor, was then coinfused with l‐NMMA at doses of 90 to 540 ng/min and titrated to restore basal blood flow. Once basal blood flow was restored, the SNP infusion was continued for the remainder of the study to produce an “NO clamp” as described previously.^24^ This permits the assessment of vascular function in the absence of endogenous NO generation but without the potential confounding effects of basal vasoconstriction induced by isolated NO synthase inhibition.

The dose‐response relationship of the vasodilators acetylcholine (5, 10, and 20 μg/min^6^; endothelium‐dependent NO donor) and SNP (2, 4, and 8 μg/min^6^; endothelium‐independent NO donor) were assessed in the presence of the clamp. The vasodilators were given in a random order and were separated by an infusion of 0.9% saline for 20 minutes. The combined total infusion rate of the parallel infusions was kept constant at 1 mL/min throughout the study.

### Study 2: Systemic Nitric Oxide Synthase Inhibition

Fifteen subjects were recruited into this study. Subjects had a 20‐gauge arterial line inserted to the radial artery of the non‐dominant arm (Leader‐Cath, Vygon SA). The arterial catheter was attached to a validated pressure transducer (Deltran^®^ II, Utah Medical Products) and data were recorded in real‐time using Powerlab instruments and LabChart software (AD Instruments Pty Ltd). A validated semi‐automatic oscillometric sphygmomanometer was placed around the upper arm of the dominant arm. Cardiac output, peripheral resistance, and stroke index were recorded using thoracic bioimpedence (Hotman^®^, Hemo Sapiens, Inc) and central arterial stiffness (pulse‐wave velocity and pulse‐wave analysis) recorded using the Vicorder (Skidmore Medical) and SphygmoCor™ apparatus (AtCor Medical).

After lying supine at rest for at least 20 minutes, l‐NMMA was infused intravenously over 5 minutes (total dose 3 mg/kg)^26^ via a 17‐gauge intravenous cannula inserted into a large antecubital vein of the dominant arm, and hemodynamic measurements were recorded for the following 45 minutes.

### Biochemical Analyses

Blood samples were analyzed for total and differential cell counts by an auto analyzer (Sysmex XE2100, Sysmex Europe GmbH). Plasma samples were collected into ethylene diamine tetra‐acetic acid and kept on ice until centrifuged at 2000*g* for 30 minutes at 4°C. Serum samples were collected and left to clot on melting ice for 60 minutes before being centrifuged at 2000*g* for 10 minutes at 4°C. Samples were immediately frozen and stored at −80°C prior to subsequent analysis.

Plasma was analyzed for the endogenous NOS inhibitors asymmetric dimethylarginine (ADMA) and symmetric dimethylarginine (SDMA) as well as the NO precursors l‐arginine and l‐homoarginine using a high‐performance liquid chromatography (HPLC) method as described previously.^27^ Briefly, arginine, homoarginine, ADMA, and SDMA were extracted from plasma using Isolute PRS cation exchange solid phase extraction columns (1 mL/50 mg; Kinesis Ltd). The columns were consecutively washed with borate buffer (1 mL), water (3 mL), and methanol (3 mL) before analytes were eluted with 3 mL of a solution containing 50% methanol and 10% concentrated ammonia in water. The eluent was then evaporated to dryness at 80°C under air. The dried extract was then dissolved in 0.1 mL water and 0.1 mL of the derivization reagent (10 mg of *ortho*‐phthaldialdehyde dissolved in 0.2 mL methanol followed by the addition of 1.8 mL of 200 nmol/L borate buffer and 10 μL of 3‐mercaptopropionic acid, then diluted 5‐fold using 200 nmol/L borate buffer immediately before use) before being transferred to autosampler vials, maintained at 10°C, and 20 μL was injected into the HPLC analytical column for chromatography. Following separation, arginine, homoarginine, ADMA, and SDMA were measured fluorimetrically (Waters 2475, Waters) and quantified using single‐level calibration using an internal standard (80 μL of 5 μmol/L monoethylarginine) added before sample extraction.

Blood samples obtained for the measurement of plasma nitrite were collected into lithium heparin, immediately transferred to Eppendorf^®^ tubes prewashed with Milli‐Q deionized water, and immediately centrifuged at 5000*g* for 1 minute. Plasma was then transferred into a dark colored Eppendorf^®^ tube containing 100 μL of a solution containing 1 mmol/L diethylenetriamine‐pentaacetic acid and 62.5 mmol/L N‐ethylmaleimide before being snap‐frozen on dry ice and stored at −80°C prior to further analysis.

Plasma nitrite samples were defrosted on ice before 100 μL was injected into a reaction vial containing glacial acetic acid and iodide.^28^ NO generated in the reaction chamber was driven off by a continual stream of oxygen‐free nitrogen and detected in the exhaust gas using a Sievers NOA 280i chemiluminescent analyzer (Analytix, Co). Analyses were performed in triplicate. The limit of detection of this assay was ≈30 nmol/L with a coefficient of variation of 3.1% for a 250 nmol/L standard.

### Data Analysis and Statistics

Plethysmography data were analyzed as described previously.^29–30^ Data are expressed as mean±standard deviation (SD) unless otherwise stated. Statistical analyses were performed using Student's paired *t*‐tests and 2‐way analysis of variance (ANOVA) with repeated measures where appropriate. Statistical significance was taken as 2‐sided *P*<0.05. All analyses were performed using GraphPad Prism (Version 4 for Macintosh, GraphPad Software) on a Macintosh personal computer.

## Results

Thirty subjects completed the study protocols (Table 1) with 5 subjects common to both protocols. All studies were generally well tolerated with no significant adverse events. There were no changes in any indices of lung function (FEV_1_, FVC, and VC) or in hemoglobin concentration, white cell count (or differential cell counts) or platelet count throughout all studies (*P*>0.05 for all; data not shown).

**Table 1.** Baseline Characteristics of Study Participants

Baseline Characteristics	Study 1: Forearm Study (n=16)	Study 2: Systemic l‐NMMA (n=14)
Age, y (median, range)	23 (21 to 27)	26 (23 to 34)
Male/Female	9 (56%)/7 (44%)	8 (57%)/6 (43%)
Use of oral contraceptive (female)	5 (72%)	3 (50%)
Height, cm		
All	176±9	178±11
Male	181±7	186±6
Female	169±6	167±5
Weight, kg		
All	72±13	74±14
Male	80±11	85±6
Female	60±4	60±3
BMI, kg/m^2^		
All	23.1±2.8	23.2±2.1
Male	24.6±2.8	24.6±1.4
Female	21.2±1.0	21.5±1.5

Data expressed as mean±standard deviation unless otherwise stated. l‐NMMA indicates N^G^‐monomethyl‐l‐arginine; BMI, body mass index.

### Plasma Nitrite and Arginine Concentrations

Two hours following exposure to diesel exhaust, plasma nitrite concentrations were higher as compared to the filtered air exposure (68±48 versus 41±32 nmol/L; *P*=0.006). In contrast, there were no differences in plasma concentrations of the NO precursor l‐homoarginine, or in the endogenous NOS inhibitors ADMA and SDMA at 2 or 6 hour following exposures (Table 2). Plasma concentrations of the NO precursor l‐arginine were lower after exposure to diesel exhaust as compared to filtered air (*P*=0.043).

**Table 2.** Plasma Concentrations of NOS Substrates and Endogenous NOS Inhibitors

Parameter	Exposure	Baseline	2 Hour	6 Hour	*P* value	
					Time	Exp
l‐Arginine, μmol/L	Air	74.1±16.3	61.6±17.8	59.3±10.6	0.006	0.006
	Diesel	67.7±13.4	58.2±10.0	54.6±10.3		
l‐Homoarginine, μmol/L	Air	1.75±0.46	1.66±0.48	1.70±0.51	0.913	0.412
	Diesel	1.67±0.51	1.63±0.48	1.69±0.53		
ADMA, μmol/L	Air	0.52±0.08	0.44±0.09	0.51±0.07	0.010	0.374
	Diesel	0.51±0.05	0.44±0.06	0.50±0.07		
SDMA, μmol/L	Air	0.43±0.06	0.41±0.07	0.43±0.05	0.451	0.449
	Diesel	0.41±0.05	0.41±0.05	0.43±0.05		

Plasma concentrations of endogenous NOS inhibitors and NOS substrates at baseline, 2 and 6 hours after the exposure during Study 1 (local l‐NMMA administration). All data expressed as mean±standard deviation. *P*‐values from 2‐way repeated measures ANOVA. NOS indicates nitric oxide synthase; ADMA, asymmetric dimethylarginine; SDMA, symmetric dimethylarginine.

### Study 1: Local Nitric Oxide Synthase Inhibition

Sixteen subjects, median age 23 years (Table 1), completed the study protocol: one subject failed to attend scheduled visits and one was withdrawn for technical reasons (failed cannulation). Blood pressure, heart rate, and baseline forearm blood flow were not different following either exposure (*P*>0.05 for all; data not shown).

**Figure 1.**
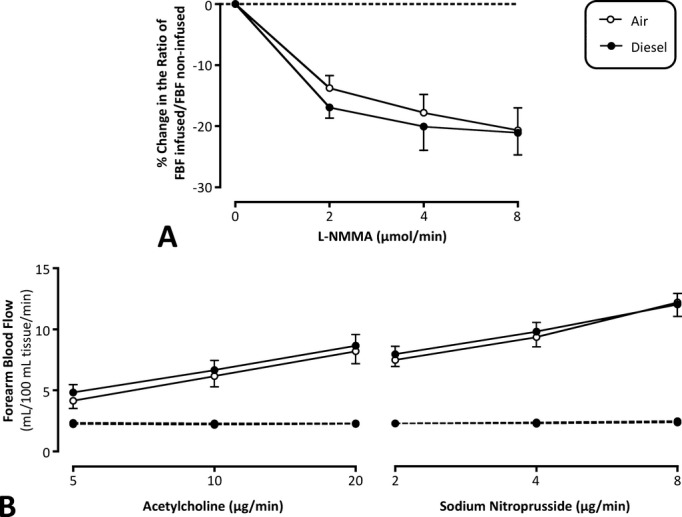
A, Forearm blood flow response during infusion of l‐NMMA (2‐way repeated measures ANOVA:* P*=0.559 for exposure). B, Forearm blood flow during infusion of the vasodilators acetylcholine and sodium nitroprusside (2‐way repeated measures ANOVA:* P*=0.209 and *P*=0.613, respectively, for exposure). Data expressed as mean±SEM. l‐NMMA indicates N^G^‐monomethyl‐l‐arginine; ANOVA, analysis of variance; SEM, standard error of the mean.

Infusion of the NOS inhibitor, l‐NMMA, resulted in a similar dose‐dependent vasoconstriction following both exposures (*P*=0.559; Figure 1A). After establishing the NO clamp by restoring basal blood flow with SNP coinfusion (208±33 and 236±26 ng/min following air and diesel exhaust exposure respectively; *P*=0.453), both acetylcholine and SNP caused dose‐dependent vasodilatation (*P*<0.01 for both) that was attenuated in comparison to previous studies.^6^ However, this vasodilatation was similar following both exposures (dilute diesel exhaust versus filter air: *P*=0.209 for acetylcholine and *P*=0.613 for sodium nitroprusside; Figure 1B).

### Study 2: Systemic Nitric Oxide Synthase Inhibition

Fourteen subjects, median age 26 years (Table 1), completed the study protocol: one subject was withdrawn for technical reasons (failed cannulation). There were no differences in hemodynamic or biochemical indices at baseline prior to either exposure (Table 3).

**Table 3.** Baseline Hemodynamic and Biochemical Parameters Prior to Exposure for the Systemic Administration of l‐NMMA Study

Parameter	Air	Diesel
Heart rate, bpm	67±13	65±8
Systolic blood pressure, mm Hg	124±12	124±16
Diastolic blood pressure, mm Hg	70±6	70±6
Mean arterial pressure, mm Hg	88±6	88±8
Pulse wave velocity, m/s	5.8±0.6	5.8±0.5
Augmentation index (AIx), %	−3±9	1±10
AIx75, %	−8±9	−6±11
Augmentation pressure, mm Hg	−1±4	0±4
Time to wave reflection, ms	164±20	172±38
Cardiac index, L/min per m^2^	4.8±0.8	5.0±0.8
Stroke index, mL/beat per m^2^	76±12	81±17
PVRI, dynes.min/cm^5^ per m^2^	91±20	88±26
Hemoglobin, g/dL	144±14	140±12
Hematocrit, %	41±2	41±3
White cell count, ×10^9^/L	5.9±0.9	5.5±0.8
Neutrophil count, ×10^9^/L	3.1±0.8	2.7±0.7
Monocyte count, ×10^9^/L	0.5±0.2	0.5±0.1
Lymphocyte count, ×10^9^/L	2.0±0.3	2.1±0.4
Platelet count, ×10^9^/L	224±38	226±33
Blood urea nitrogen, mg/dL	16.8±7.6	16.8±7.6
Creatinine, mg/dL	0.85±0.16	0.85±0.16
Sodium, mmol/L	141±1	141±1
Potassium, mmol/L	4.3±0.3	4.2±0.3
TCO_2_, mmol/L	28±3	28±2
eGFR, mL/min per 1.73 m^2^	121±26	122±26

Data expressed as mean±standard deviation. l‐NMMA indicates N^G^‐monomethyl‐l‐arginine; bpm, beats per minute; AIx, augmentation index corrected for a heart rate of 75 bpm; PVRI, peripheral vascular resistance index; TCO_2_, total carbon dioxide; eGFR, estimated glomerular filtration rate.

**Figure 2.**
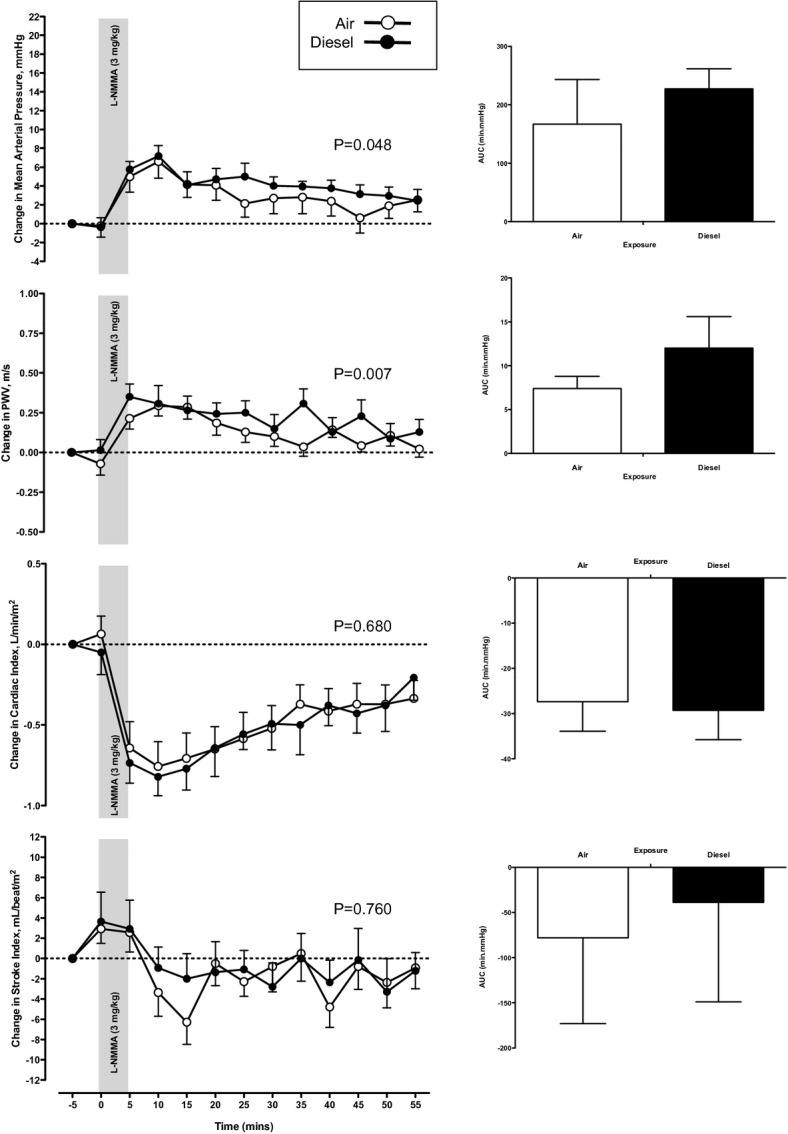
Changes in invasive mean arterial blood pressure, central arterial stiffness (PWV), cardiac index, and stroke volume following systemic infusion of l‐NMMA. Data expressed as mean±SEM and composite area under the curve with 2‐way repeated measures ANOVA for exposure. PWV indicates pulse‐wave velocity; l‐NMMA, N^G^‐monomethyl‐l‐arginine; SEM, standard error of the mean; ANOVA, analysis of variance.

Following exposure to diesel exhaust, l‐NMMA caused a greater increase in blood pressure (*P*=0.048) and central arterial stiffness (*P*=0.007) with similar reductions in cardiac output and increases in systemic vascular resistance (*P*>0.05 for both) as compared to filtered air (Figure 2).

## Discussion

We have demonstrated that inhalation of dilute diesel exhaust increases plasma nitrite concentrations suggesting an increase in basal NO release. However, local NO synthase inhibition causes similar degrees of vasoconstriction following both diesel exhaust and filtered air exposure. This suggests a balanced increase in basal NO generation and consumption that attempts to maintain basal peripheral resistance vessel tone. This is consistent with our observation that diesel exhaust inhalation‐induced vasomotor dysfunction^31^ is no longer demonstrable in the presence of the NO clamp. In contrast to the peripheral resistance vessel responses, systemic NO synthase inhibition demonstrated an increase in systemic arterial stiffness and blood pressure suggesting that, in conduit vessels, reduced NO bioavailability has more marked effects that cannot be adequately compensated for by increased basal NO release.

We demonstrated higher basal venous plasma nitrite concentrations following diesel exhaust exposure. These data are consistent with the upregulation of vascular NO generation, which we suggest may represent a homeostatic feedback loop to compensate for increased consumption by local oxidative stress in order to maintain basal vascular tone and blood pressure. While it is unlikely that changes in NO generation reflect upregulation of gene expression, there is growing evidence that endothelial NOS (eNOS) can be rapidly upregulated or induced by enzyme phosphorylation and alterations in protein–protein interactions^32^ and we suggest that these interactions are responsible for the increased NO generation due to local inflammation and oxidative stress. Indeed, these interactions occur very rapidly in animal models, with LPS‐induced inflammation increasing eNOS activity almost 2‐fold within 1 hour.^33^ We believe this effect is likely to be driven by changes in eNOS activity although the exact isoform of the NOS enzyme responsible is unclear from our study given the nonselective inhibition by l‐NMMA. Previous studies in murine models have demonstrated an increase in inducible‐NOS (iNOS) expression following exposure to dilute diesel exhaust,^34–35^ and certainly iNOS has the capacity to be rapidly upregulated in response to insult. The possibility of an effect on iNOS expression remains unexplored at this time.

We have previously demonstrated in isolated rat aortic rings that diesel exhaust particles attenuate endothelium‐derived vasorelaxation. This effect was reversed by coincubation with superoxide dismutase thereby reducing oxygen‐centered free radical production.^36^ We have also shown that diesel exhaust particles themselves reduce the concentrations of bioavailable NO from the NO donor DEA/NO (2‐(N,N‐diethylamino)‐diazenolate‐2‐oxide).^19^ Nurkiewicz and colleagues have reported that, following inhalation of titanium dioxide nanoparticles, local NO was scavenged by oxygen‐centered free radicals.^20^ This reduction in NO bioavailability appears to be driven either by the uncoupling of endothelial NOS by diesel exhaust particles or by local oxidative stress.^37^ However, while reflective of production and consumption of NO across the vascular bed, plasma nitrite concentrations do not definitively describe the activity of the NOS enzymes and their isoforms. It remains feasible that some of the increase in plasma nitrite is in fact due to absorption from the higher oxides of nitrogen during the exposure itself. Knuckles and colleagues have recently reported that following diesel exhaust exposure, rats had increased plasma NO_x_ (nitrate and nitrite combined) concentrations that appeared to be predominantly the result of absorption of NO from the exposure itself, although they exhibited significant increases in eNOS expression consistent with increased NO production.^38^ Controlled exposures to NO and nitrogen dioxide in humans with subsequent measurements of plasma nitrite concentrations may help to define this further.

The concept of local oxidative stress is thought to underlie vascular endothelial dysfunction in clinical states such as hypertension, diabetes mellitus, and hypercholesterolemia,^39^ and has been proposed as the central mechanism linking the cardiovascular, respiratory, and inflammatory responses to inhaled particulate air pollution.^40^ Measurement of oxidative stress is, however, extremely difficult to quantify due to the short half‐life and high reactivity of free radicals, and because we are largely left to measure surrogate markers such as isoprostanes and oxidative DNA adducts to determine increases in free radical production in clinical models. As such, the clinical evidence for increased oxidative stress following exposure to air pollutants is limited, and existing studies are inconsistent, although a recently published meta‐analysis suggests that there is a robust relationship between exposure to particulate air pollution and increases in oxidative DNA adducts and oxidized lipids in man.^41^ While outside of the scope of this study, we have previously measured plasma nitrotyrosines and urinary isoprostanes following exposure to dilute diesel exhaust, and these results are presented in an online supplement (Data S1).

Although the plasma concentration of the NO precursor l‐arginine was generally lower after exposure to diesel exhaust, plasma concentrations of l‐homoarginine were unaffected and there were no changes in concentration over time. These precursors, l‐arginine and l‐homoarginine, are present in vast molar excess as only 1.2% of plasma arginine used for whole‐body NO production.^42–43^ The small differences in plasma concentration, which are most likely attributable to dietary intake, are of little functional significance and cannot account for differences in NO production or availability. Similarly, given the small amount of l‐arginine/l‐homoarginine required to produce NO, we suggest short‐term modest increases in NO generation are unlikely to affect their plasma concentrations or indeed those of the naturally occurring endogenous NOS inhibitors, such as ADMA.

Individuals with hypercholesterolemia have impaired vascular vasomotor dysfunction: the classic paradigm of vascular endothelial dysfunction as a result of changes in the l‐arginine‐NO pathway.^44–45^ However, these subjects have normal responses to NOS inhibition with l‐NMMA, but reduced vasodilatation to endothelium‐dependent vasodilators such as acetylcholine and serotonin.^46^ In the present studies, we demonstrate the same pattern of vascular dysfunction following the inhalation of dilute diesel exhaust. Indeed, in the presence of the NO clamp, and the absence of endogenous NO release, the previously demonstrated attenuation in vascular vasomotor responses following diesel exhaust inhalation^31^ is abolished. These findings are consistent with the suggestion that basal NO generation is already increased so further stimulation may be proportionately less effective, and that the NO released may be consumed more rapidly by local oxidative stress. Furthermore, given that the responses to stimulation with acetylcholine are similar in the absence of endogenous NO release, this would suggest that the other key vasodilator mechanisms, such as endothelium‐derived hyperpolarising factor (EDHF) or prostaglandins, are intact and unaffected by diesel exhaust inhalation.

Administration of the exogenous NOS inhibitor, l‐NMMA, had the anticipated response of causing peripheral vasoconstriction and a systemic pressor response with reduced cardiac output.^26,47–51^ The control of basal vascular tone and vascular reactivity is complex and is known to vary in the different vascular beds. The regulation of conduit arterial tone is almost solely dependent on endothelial NO production, and alternative vasodilator mechanisms, such as EDHF, appear to play little role.^52^ In contrast, the vasomotor responses in resistance vessels are mediated through NO, EDHF, and prostaglandin pathways.^53–54^ Indeed, endogenous NO release reflects only lower levels of vasodilatation, with EDHF compensating and providing a greater contribution to the vasomotor response especially with higher degrees of vasodilatation. We therefore suggest that in the conduit vessels, the increase in endogenous NO production following diesel exhaust inhalation can offset the increased consumption of NO in the context of increased local oxidative stress, thus suppressing a potential pressor effect. However, in the presence of systemic NOS inhibition, this potential pressor effect is unmasked and we see an increase in blood pressure and arterial stiffness. This enhanced sensitivity of the central arterial tree may underlie the increases in blood pressure seen with acute^8,55^ and chronic^56^ exposures to particulate air pollutants. Alternatively, the increase in NO concentrations following exposure to diesel exhaust may reflect increased bioavailability of NO within the vasculature, and an enhanced pressor response to systemic l‐NMMA infusion. However, this would not be consistent with the similar basal blood flow and vasomotor response to l‐NMMA in the forearm circulation.

While we suggest that changes in NO bioavailability are consistent with our findings, a similar effect may be seen with other mechanisms, such as activation of the autonomic nervous system. Such activation will also cause an increase in vascular tone as well as a pressor and arterial stiffness response. Indeed, changes in autonomic nervous system activation may underlie the acute vasoconstriction^7^ and changes in heart rate variability previously reported.^57^ Further studies are warranted to evaluate the contribution of the autonomic nervous system to these important vascular effects.

### Limitations

This study was undertaken in order to examine changes in blood pressure and arterial vasomotor function as measured by venous occlusion plethysmography. While the robust crossover design increases the statistical power of the study, we recognize that for some secondary outcomes this study may have been unable to definitively identify a lack of effect.

## Conclusions

In these studies we have demonstrated for the first time in humans that inhalation of dilute diesel exhaust results in higher basal plasma nitrite concentrations. This is consistent with homeostatic regulation in the presence of increased NO consumption due to local oxidative stress. Similarly, we have shown that in the presence of the NO clamp and the absence of endogenous NO production, diesel exhaust inhalation does not result in further vascular impairment. Furthermore, diesel exhaust inhalation exaggerates the pressor and vasoconstrictor effects of systemic NO synthase inhibition consistent with the proposed changes in NO production and consumption. We conclude that the vascular dysfunction associated with diesel exhaust inhalation is mediated predominantly by reduced NO bioavailability.
